# Improvement of ligand-free modification strategy to obtain water-stable up-converting nanoparticles with bright emission and high reaction yield

**DOI:** 10.1038/s41598-021-98240-0

**Published:** 2021-09-22

**Authors:** Natalia Jurga, Dominika Przybylska, Piotr Kamiński, Tomasz Grzyb

**Affiliations:** Department of Rare Earths, Faculty of Chemistry, Adam Mickiewicz University in Poznań, Uniwersytetu Poznańskiego 8, 61-614 Poznan, Poland

**Keywords:** Nanoparticles, Nanoscale materials, Materials chemistry, Optical materials, Optical materials and structures, Nonlinear optics, Synthesis and processing

## Abstract

Water-dispersible up-converting nanoparticles (UCNPs) are known to be very effective in biomedical applications. Research groups have paid special attention to the synthesis of hydrophilic UCNPs with good physicochemical properties. Being aware of this, we decided to improve the ligand-free modification method of OA-capped NaYF_4_:Yb^3+^,Er^3+^/NaYF_4_ UCNPs prepared by precipitation in high-boiling-point solvents as the thus-far reported methods do not provide satisfactory results. Different molarities of hydrochloric acid and various mixing times were selected to remove the organic ligand from the NPs’ surface and to discover the most promising modification approach. Highly water-stable colloids were obtained with a very high reaction yield of up to 96%. Moreover, the acid treatment did not affect the morphology and the size of the product. All of the crystals exhibited a bright up-conversion emission under 975-nm excitation, which confirmed the two-photon excitation and effective energy transfer between the used dopant ions. Thus, we could establish the most successful ligand-free modification procedure.

## Introduction

The lanthanide (Ln^3+^)-doped up-converting nanoparticles (UCNPs) are interesting materials because of their specific optical properties, i.e., long luminescence lifetimes and the sharp emission bands related to the Ln^3+^ electronic configuration^1^. In the up-conversion (UC) process, the absorption of two or more near-infrared (NIR) photons is followed by the emission of light in the UV and the visible range^2^. The system consisting of two dopant ions Yb^3+^/Er^3+^ is perfect to study one of the UC mechanisms called energy transfer, in which the energy is absorbed by the sensitizer (Yb^3+^ ions) and transferred to the activator (Er^3+^ ions)^3^. By changing the dopant-ion combination and concentration, we can observe the multi-color UC emission^4,5^. Note that using NIR light as an excitation source is beneficial because of the deep penetration depth in tissues, small photodamage to living organisms, and the absence of autofluorescence from cells^1,6^.

The appropriate selection of UCNPs’ host matrix plays an important role in achieving the desired luminescent properties^7^. The commonly used hosts of the UC materials are fluorides because of their excellent chemical stability and low phonon energy. The obtainment of Ln^3+^-doped NaYF_4_ with a hexagonal structure is particularly beneficial, as it is considered to be the most profitable UC luminophore^8^. Additionally, the UC efficiency can be enhanced by the presence of the inert shell, which protects luminescent ions in the core from the non-radiative transitions^9^. The high-quality UCNPs with a particular size, shape, and phase can be synthesized using precipitation in the high-boiling-point solvent method. During the synthesis, selected rare earth (RE) ion precursors are decomposed to form the desired UCNPs by controlling the temperature, pressure, and solvent composition^10^. Unfortunately, the synthesized oleate-capped (OA-capped) UCNPs cannot be used in tissue and cell experiments because of their insolubility in water^11^. Therefore, it is advantageous to modify their surface^12,13^.

Water is the main component of living organisms. Therefore, the hydrophilic NPs are extremely attractive candidates for biomedical applications such as bioimaging, photodynamic therapy, drug delivery, cellular nanothermometry, harmful ion sensors, and cancer diagnosis and treatment^14–20^. Regrettably, the NPs dispersed in water show decreased luminescence intensity as compared to those dispersed in organic solvents. This phenomenon is observed because of the quenching related to the high vibrational levels of hydroxyl groups^21^.

It is possible to distinguish several strategies of modifying the NP surface, such as layer-by-layer assembly, ligand exchange, ligand oxidation, ligand-free modification, and silanization^22^. The ligand-free modification method of the NP surface described by Capobianco’s research group is well known and used^23^. This method allows the preparation of water-dispersible NaYF_4_ doped with Ln^3+^ ions with high UC emission intensity. As a result of the acid treatment (pH = 4), the oleate ligands are removed from the surface of the OA-capped UCNPs. The modified NPs are stable in aqueous solutions and ready for further surface conjugation with biomolecules^22,24^. This approach can also be used in other host materials^18,25,26^. Thus far, many research groups have obtained ligand-free materials according to the described procedure, often making small changes. For instance, Guo et al*.* and Liu et al*.* decided to change the molarity of the used hydrochloric acid^21,27^. It is worth mentioning that pH values are selected depending on the particle size, e.g. pH < 3 are reported for β-phase crystals smaller than 30 nm^27^. Other research groups changed the ultrasonication time, as well as the time of mixing the NPs with acid^20,28,29^. Note that the described method can be associated with difficulties, such as the tendency of NPs to agglomerate. Therefore, the yield of this modification is usually only in the range of 30–35%^24^. Moreover, the acidic environment can lead to α-NaREF_4_ dissolution followed by precipitation of REF_3_. Fortunately, the phase transformation is not observed for the thermodynamically stable β-phase NaREF_4_^27^.

The main goal of our study was to improve the ligand-free modification method of OA-capped UCNPs synthesized using precipitation in high-boiling-point solvents. We developed a novel procedure to obtain bare core/shell NaYF_4_:Yb^3+^,Er^3+^/NaYF_4_ UCNPs with a high reaction yield of up to 96%. Different molarities of hydrochloric acid and various reaction times were used to transfer the product from an organic to an aqueous solution: 2 M/2 h, 0.1 M/2 h, and 2 M/15 min, respectively. The effects of the changing modification conditions on the reaction yield, UCNP morphology, and spectroscopic properties were demonstrated. Furthermore, the stability of water-dispersible UC nanomaterials was confirmed.

## Materials and methods

### Materials

The RE oxides Y_2_O_3_ (99.99%), Er_2_O_3_ (99.99%), and Yb_2_O_3_ (99.99%) were purchased from Stanford Materials. Ethanol (99.8%), acetic acid glacial (99.5%), and acetic acid (80%) were obtained from POCH S.A; oleic acid (90%) and 1-octadecene (90%) from Alfa Aesar; and n-hexane (≥ 99%) from Honeywell. Hydrochloric acid (ultrapure, 37%) and sodium oleate (82%) were obtained from Sigma-Aldrich and ammonium fluoride (99%) from ACS Reagent. The synthesis of NPs was carried out under the flow of nitrogen (99.99%, Linde). In the surface modification process, hydrochloric acid (ultrapure) from Romil was used. The aqua solutions of hydrochloric acid were prepared by the dissolution of the above-mentioned ultrapure hydrochloric acid in distilled water.

### Synthesis of OA-capped NaYF_4_:Yb^3+^,Er^3+^/NaYF_4_ core/shell up-converting nanoparticles

The core–shell NaYF_4_:Yb^3+^,Er^3+^/NaYF_4_ nanomaterials were synthesized using precipitation in high-boiling-point solvents^30,31^. Two different RE ion precursors (RE acetates and RE chlorides) were used to obtain materials with the desired size. The approach was based on the heating of RE ion precursors in the mixture of high-boiling-point solvents oleic acid and 1-octadecene (1:1 ratio), after their earlier outgassing under low pressure (< 10^−1^ mbar). A detailed description of the procedure is presented in Supporting Information.

### Synthesis of ligand-free NaYF_4_:Yb^3+^,Er^3+^/NaYF_4_ core/shell up-converting nanoparticles

**Figure 1:**
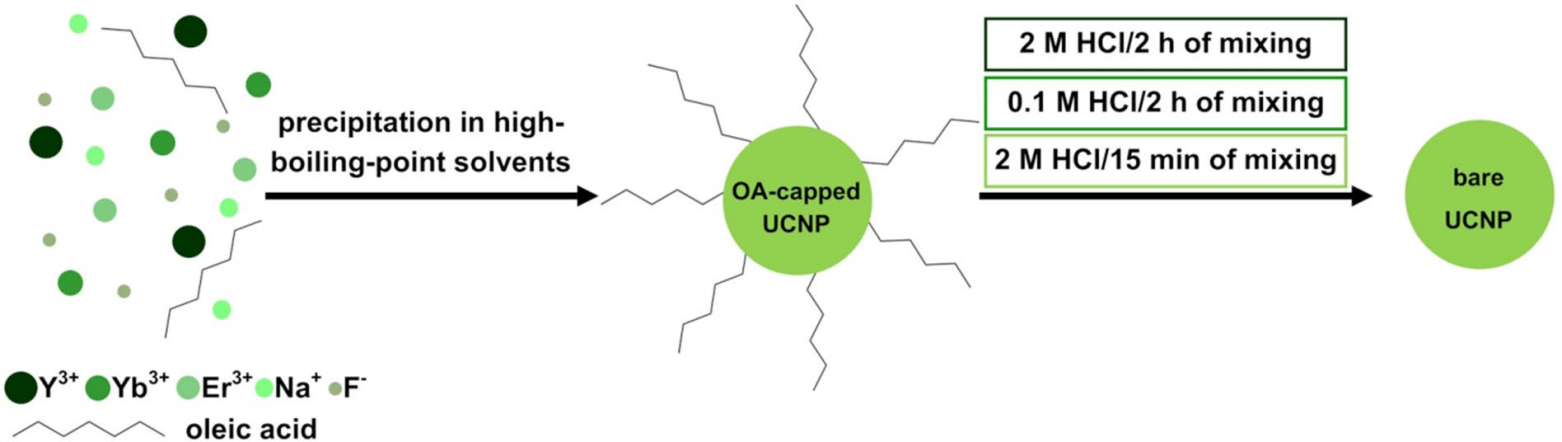
Schematic representation of the synthesis and ligand-free modification of OA-capped NaYF_4_:Yb^3+^,Er^3+^/NaYF_4_.

The ligand-free NaYF_4_:Yb^3+^,Er^3+^/NaYF_4_ NPs were prepared through the acidic treatment of the OA-capped UCNPs by using the procedure reported by Bogdan et al*.* with some modifications^23^. Firstly, 100 mg of the appropriate OA-capped NPs were dispersed in 5 mL of n-hexane, resulting in the NPs concentration of 20 mg/mL. To this suspension, 2.5 mL of the water solution of hydrochloric acid (2 M or 0.1 M) was added to protonate the oleate ligands, which led to removal of the oleic acid from the NPs’ surface. The flasks were closed, and the mixtures were vigorously stirred at room temperature (2 h or 15 min). On the basis of a series of laboratory experiments, three different combinations of HCl molarity and mixing time were chosen and reported in this article: 2 M/2 h, 0.1 M/2 h, and 2 M/15 min, respectively. Note that the reaction time in the last experiment was decreased to 15 min because after that time, the organic solvent became transparent and the DLS analysis confirmed the absence of UCNPs in the organic phase. Therefore, it was assumed that the particles were successfully transferred to the aqueous solution. Next, the mixtures were ultrasonicated for 5 min and transferred to a centrifuge tube. The products were collected by centrifugation at 9000 rpm for 15 min and washed with a mixture of water and ethanol (in the ratio of 1:1) to discard organic layer containing the oleic acid molecules. The washing procedure was repeated two times. The bare UCNPs were dispersed in distilled water and stored at 4 °C. A schematic representation of the synthesis and surface modification of OA-capped UCNPs is presented in Fig. 1.

### Characterization of materials and instrumentation

The structure of OA-capped nanomaterials was characterized by an X-ray diffraction (XRD) powder measurement using a Bruker AXS D8 Advance X-ray diffractometer with Cu Kα radiation (λ = 0.15406 nm). The morphology and the size of the products were determined by transmission electron microscopy (TEM) using an HT7700 electron microscope operating at 40 or 120 kV. The hydrodynamic diameter, polydispersity index and the zeta potential of UCNPs were measured in n-hexane (OA-capped NPs; 0.25 mg/mL) and the water solution (bare NPs; 0.25 mg/mL) at 25 °C by Zetasizer Nano ZS from Malvern equipped with a laser with 632.8-nm wavelength and using backward scattering (173°). A thermogravimetric (TGA) analysis was performed using Thermogravimetric Analyzer TGA 4000 (Perkin Elmer). The measurement was conducted in the temperature range of 30–600 °C (heating rate: 10 °C/min). Infrared spectra were registered for samples in KBr pellets using JASCO 4200 FT-IR spectrophotometer. The spectroscopic properties of the OA-capped nanomaterials dispersed in n-hexane (1 mg/mL) and the bare UCNPs dispersed in water (1 mg/mL) were registered at room temperature using a fiber-coupled CNI 2 W continuous wave (CW) diode laser with 808-, 975-, 1208-, and 1532-nm wavelengths equipped with PIXIS:256E Digital CCD Camera with SP-2156 Imaging Spectrograph (Princeton Instruments). The NPs were excited with the abovementioned CW laser using λ_exc_ = 975 nm.

## Results and discussion

**Figure 2:**
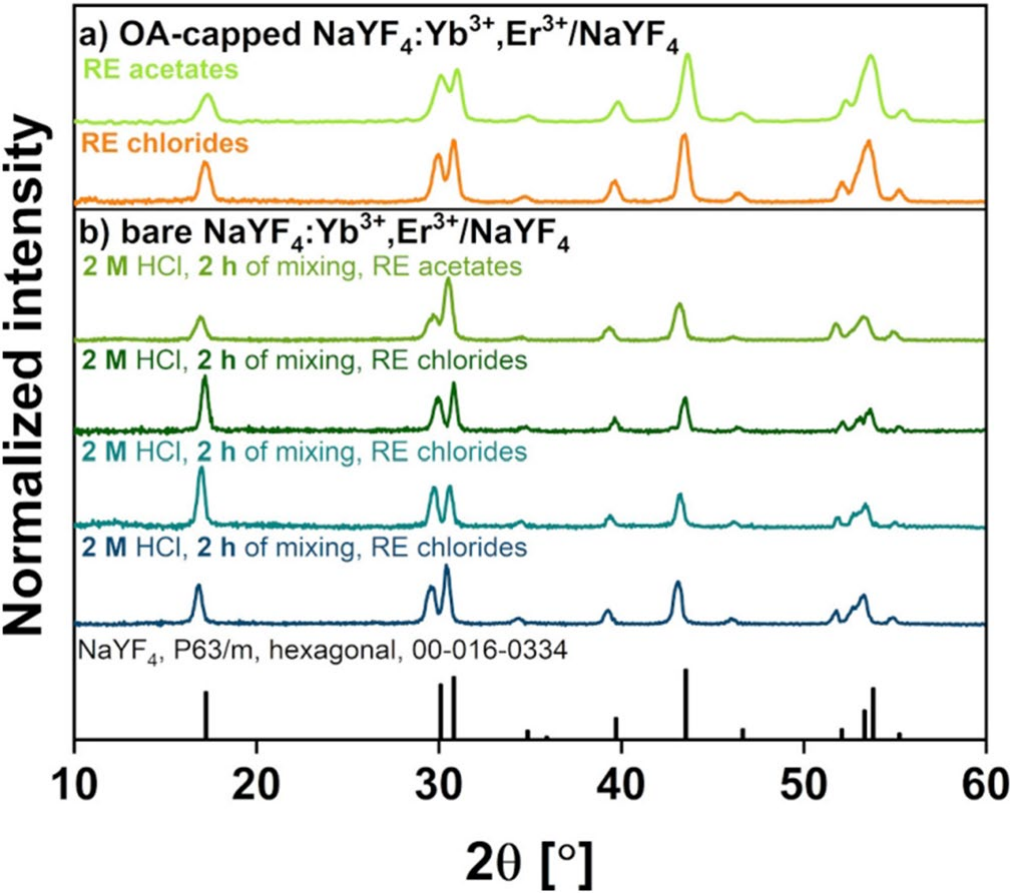
XRD patterns of (**a**) OA-capped and (**b**) ligand-free core/shell NaYF_4_:Yb^3+^,Er^3+^/NaYF_4_ UCNPs, and the XRD reference pattern from the JCPDS Database 00-016-0334.

The core/shell UCNPs were synthesized by precipitation in high-boiling-point solvents. Directly after the NPs’ synthesis, the structure and the phase purity of the OA-capped products obtained from RE acetates and RE chlorides were verified using an XRD analysis (Fig. 2). The NaYF_4_:Yb^3+^,Er^3+^/NaYF_4_ crystals exhibited a hexagonal phase, which was consistent with the reference pattern JCPDS 00–016-0334. The registered broad reflections confirmed that the products were nano-sized and well crystallized. Moreover, no additional impurity peaks were observed. The XRD analysis was also performed for the ligand-free UCNPs. Importantly, no phase and structure transformations were registered after the acid treatment of UCNPs.

**Figure 3:**
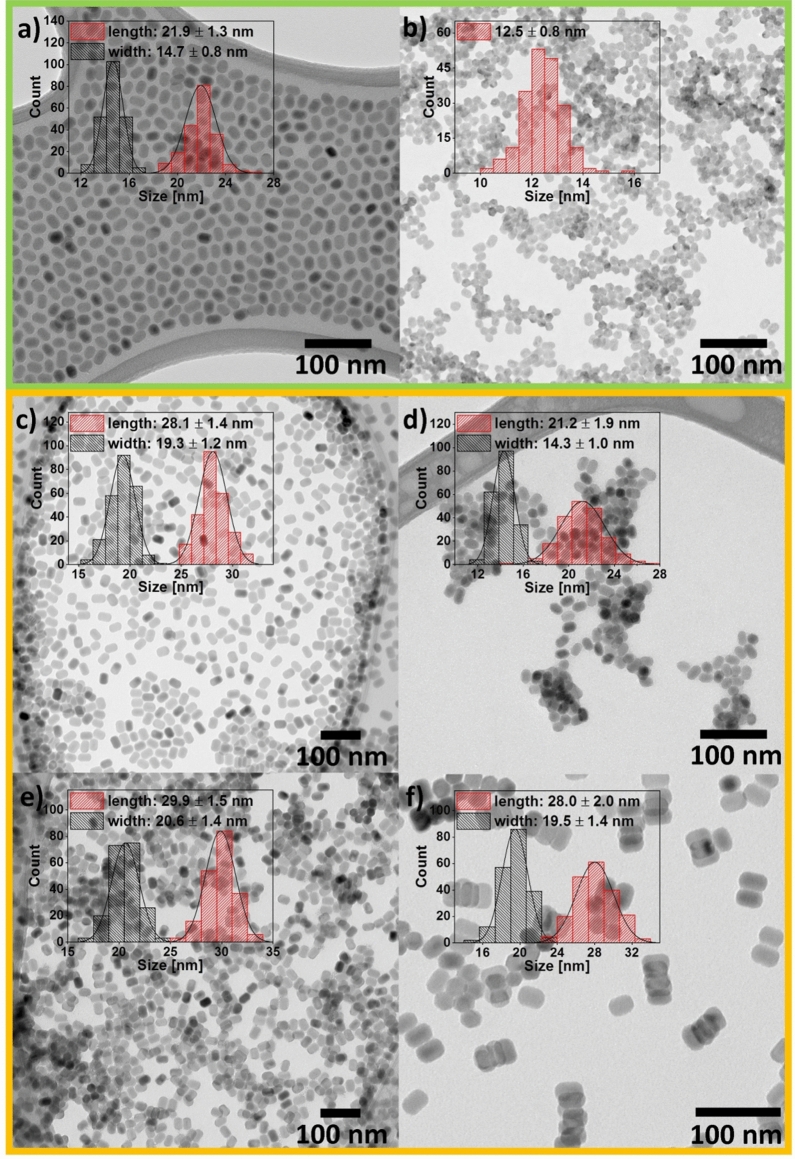
TEM images and particle size distribution of (**a**,**c**) OA-capped and (**b**,**d**,**e**,**f**) ligand-free NaYF_4_:Yb^3+^,Er^3+^/NaYF_4_ UCNPs. Different modification conditions were used (**b**,**d**) 2 M/2 h, (**e**) 0.1 M/2 h, and (**f**) 2 M/15 min, which denote the HCl molarity and the time of mixing, respectively. Color coding is used to indicate the UCNPs obtained from the RE acetates (green) and the RE chlorides (orange).

The size and the morphology of the obtained products were determined using a TEM analysis (Fig. 3). Both the OA-capped and the ligand-free NPs had an average size of less than 30 nm and a narrow size distribution, which indicated that the obtained materials were of good quality. Additionally, the UCNPs did not agglomerate even after the removal of ligands from their surface. This suggests that the positively charged bare Ln^3+^-UCNPs were surrounded by the water molecules, which prevented their agglomeration. Firstly, the materials were obtained using RE acetates. The products were in the form of small rods with 21.9 ± 1.3 nm in length and 14.7 ± 0.8 nm in width (Fig. 3a). The TEM pictures were also collected after the surface modification of the OA-capped NPs. Their average size was decreased (12.5 ± 0.8 nm), and the shape changed to hexagonal (Fig. 3b). Long-term exposure of NPs to HCl solution causes the shell etching and is usually followed by increasing number of surface defects^32^. Such weakened ligand-free NPs are more susceptible to the dissolution process, which leads to size reduction. After observing the effect of the modification process on the NPs’ morphology and size and considering that the solubility rate depends on surface-to-volume ratio^33^, it was decided to obtain the OA-capped product with a slightly larger size to conduct more experiments and better illustrate the changes. On the basis of the laboratory experience of our research group, RE chlorides were chosen as the RE ion precursors. The use of RE chlorides instead of RE acetates was more beneficial, and it allowed us to prevent the production of toxic compounds during the thermolysis of trifluoroacetate^8^. Note that the OA-capped NPs obtained from the RE chlorides maintained the same morphology as the crystals obtained from the RE acetates but had a larger size (length: 28.1 ± 1.4 nm, width: 19.3 ± 1.2 nm; Fig. 3c). Similar to the previously conducted experiment, the selected reaction conditions (2-M HCl and 2 h of mixing) reduced the size of the bare UCNPs (length: 21.2 ± 1.9 nm, width: 14.3 ± 1.0 nm; Fig. 3d) in comparison to the OA-capped crystals. To prevent this phenomenon, the molarity of the used acid and the stirring time were modified, respectively. After changing the modification conditions, the size of the bare crystals changed insignificantly, within the limits of error. The rods with a length of 29.9 ± 1.5 nm and a width of 20.6 ± 1.4 nm (Fig. 3e) were obtained with 0.1-M HCl and 2 h of stirring. In turn, the application of 2-M HCl and the reduction of the mixing time to 15 min allowed us to obtain NPs with a length of 28.0 ± 2.0 nm and a width of 19.5 ± 1.4 nm (Fig. 3f). Importantly, after the modification of the OA-capped UCNPs obtained from the RE chlorides, the morphology remained unchanged. Nevertheless, despite of the NPs size reduction, all of the bare particles retain pure hexagonal phase which was confirmed by the XRD analysis. The results show how important it is to find the appropriate mixing time that allows for the removal of ligands from the particle surface without adversely affecting the properties of the nanoparticles.

Additionally, the size distribution was determined using DLS measurements (Fig. S1). It was again confirmed that changing the RE ion precursor (from RE acetates to RE chlorides) allowed us to obtain bigger NPs. Moreover, the size of ligand-free nanomaterials was larger than that calculated from the TEM images. This was related to the fact that the DLS analysis provided information about the NPs’ hydrodynamic diameter swelling in a water solution, whereas TEM determined the size of the crystals. The polydispersity index (PdI) were determined to check the NPs’ size uniformity and samples’ heterogeneity. It is assumed that the monodisperse NPs with narrow size distribution are characterized by the PdI values lower than 0.1. When the PdI values are between 0.1 and 0.4, the particles are polydisperse with a medium size distribution. The higher PdI values indicate the presence of NPs with various sizes which is not acceptable in biomedical applications^11^. The PdI values of OA-capped crystals obtained from RE acetates and RE chlorides were in the range of 0.07–0.09, which confirmed monodispersity of NPs. The increase of PdI values of bare crystals compared to OA-capped can be observed (PdI values in the range of 0.21–0.38). However, the values still do not exceed 0.4.

The zeta potential measurements were conducted to verify the removal of the ligands from the NPs’ surface and to specify the stability of the bare nanomaterials in an aqueous solution. During UCNPs’ acid treatment, the oleate ligands are protonated and dissociated from the NPs’ surface leaving positively charged Ln^3+^-UCNPs. The water molecules, which are present in the aqueous solution, can surround the NPs and thereby improve colloid stability. Importantly, the pH of prepared water colloids was in the range of 6.3 to 6.5, which suggests the presence of H_3_O^+^ ions in the solutions. Therefore, it is possible to observe the repulsive electrostatic forces between two adjacent UCNPs with positive zeta potential values, which results in preventing crystals agglomeration. The smallest value of the zeta potential (33.1 ± 5.4 mV) had the bare NPs obtained from the RE acetates after the 2-M HCl treatment and 2 h of mixing. In the case of the ligand-free products obtained from the RE chlorides, the following zeta potential values were recorded: 39.9 ± 5.4 mV, 36.1 ± 7.1 mV, and 39.4 ± 6.0 mV for the subsequent HCl solution molarities and the times of mixture stirring: 2 M/2 h, 0.1 M/2 h, and 2 M/15 min, respectively. The surface charges of all the ligand-free products were positive, which confirmed that the NPs’ surface modification was successful. Interestingly, the zeta potential values were higher than 30 mV even after six months for all of the modified crystals. This indicated the very good stability of the NPs in the water solution.

The FT-IR measurements of both OA-capped and bare crystals were carried out to confirm the dissociation of oleate ligands from the NPs’ surface (Fig. S2). The FT-IR spectra of OA-capped particles showed two peaks at 2930 cm^−1^ and 2854 cm^−1^, which are attributed to the –CH_2_ and –CH_3_ stretching, respectively. Moreover, the presence of bands at 1467 cm^−1^ and 1557 cm^−1^ confirmed the asymmetric and symmetric –COO– stretches. After the ligands removal, the bands related to organic groups stretching vibrations were significantly weakened. The additional band appeared at 1630 cm^−1^ and is assigned to the bending mode of water molecules^34^.

The modification conditions were changed to obtain a stable suspension of NPs in water without the change in their morphology and size. However, achieving the highest possible modification yield, which resulted in the smallest product loss, was also very important to our research group. Conducting the 2-h synthesis with the use of the 2-M HCl solution allowed us to obtain a reaction yield in the range of 47–50%. Unfortunately, the modification yield decreased to 37% with the reduction of the molarity of the hydrochloric acid solution. It can be related to the fact that the 0.1-M HCl solution was not sufficiently strong to protonate all of the OA anions; hence, not all of the particles were transferred from the organic solution to water. However, the combination of the 2-M HCl solution and 15 min of mixing turned out to be the most beneficial, as it increased the reaction yield up to 96%. Several repetitions of this approach were performed, in which the reaction yields were in the range of 75–96%. Moreover, the physicochemical properties of these ligand-free NPs were satisfactory, which was proven in this article.

**Figure 4:**
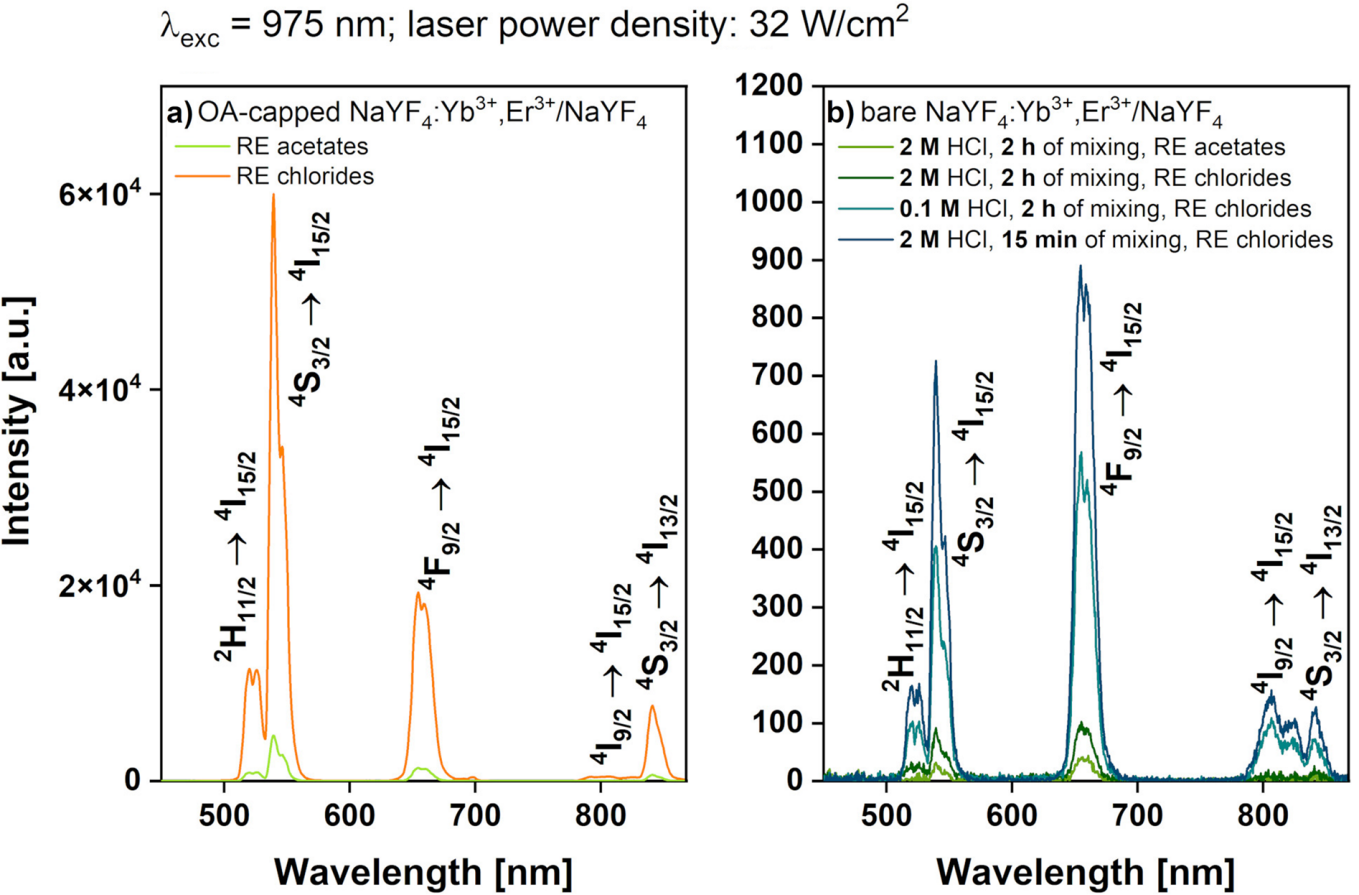
UC emission spectra of (**a**) OA-capped NaYF_4_:Yb^3+^,Er^3+^/NaYF_4_ UCNPs obtained from RE acetates and RE chlorides and (**b**) bare UCNPs after ligand-free modification with three different combinations of HCl molarity and mixing time, λ_exc_ = 975 nm. The NPs’ concentration was 1 mg/mL for both the OA-capped and the bare UCNP solutions.

The obtained materials are particularly interesting because of their unique spectroscopic properties. The UC emission spectra of the OA-capped UCNPs dispersed in n-hexane and the bare UCNPs dispersed in water were recorded using the same excitation wavelength (λ_exc_ = 975 nm), laser power density (32 W/cm^2^), and NPs’ concentration (1 mg/mL; Fig. 4). On each spectrum, we observed five narrow bands related to the Er^3+^ electronic transitions. The green emission color was strongly attributed to the transitions from the ^2^H_11/2_ and ^4^S_3/2_ to the ^4^I_15/2_ level. In turn, the transition from the ^4^F_9/2_ to the ^4^I_15/2_ level was responsible for the red luminescence color. The three bands centered at 525, 544, and 658 nm had the strongest influence on the emission color. Two other bands centered at 805 and 844 nm were distinguished; they were associated with the ^4^I_9/2_ → ^4^I_15/2_ and the ^4^S_3/2_ → ^4^I_13/2_ transitions of Er^3+^, respectively. Note that the OA-capped product synthesized from the RE chlorides was characterized by the higher luminescence intensity than that of the product obtained from the RE acetates (Fig. 4a). This fact could be related to the different sizes of NPs and the reduction of the surface defects in the larger crystals. The spectroscopic properties of the modified NPs were also determined (Fig. 4b). The emission intensity of ligand-free products considerably decreased in comparison to the OA-capped NPs. This phenomenon was connected to the non-radiative transfer of energy from the dopant ions to the water molecules. Note that of all the bare particles, the brightest luminescence had the product modified for 15 min with the 2-M HCl solution. Slightly worse spectroscopic properties had NPs, in which the ligands were removed by the 0.1-M acid for 2 h. The lowest emission intensities were recorded when the NPs were treated with 2-M HCl for a long time. Therefore, it could be concluded that with the reduction of the product size, the luminescence intensity decreased. It was confirmed that the prolonged exposure of particles to acid caused visible changes in the NPs’ size associated with the shell etching. Furthermore, the comparison of the peak area of the bands related to the green and red emission colors was shown. Interestingly, the green-to-red emission ratio was tuned from values close to 2.5 to values below 1, when the products were transferred from an organic to an aqueous solution. It showed that the UC luminescence of the OA-capped crystals was dominated by the green emission color as opposed to the emission color of the bare NPs dominated by the red. This was the effect of the non-radiative depopulation of the ^2^H_11/2_ and ^4^S_3/2_ states to the red-emitting ^4^F_9/2_ level caused by the –OH groups.

**Figure 5:**
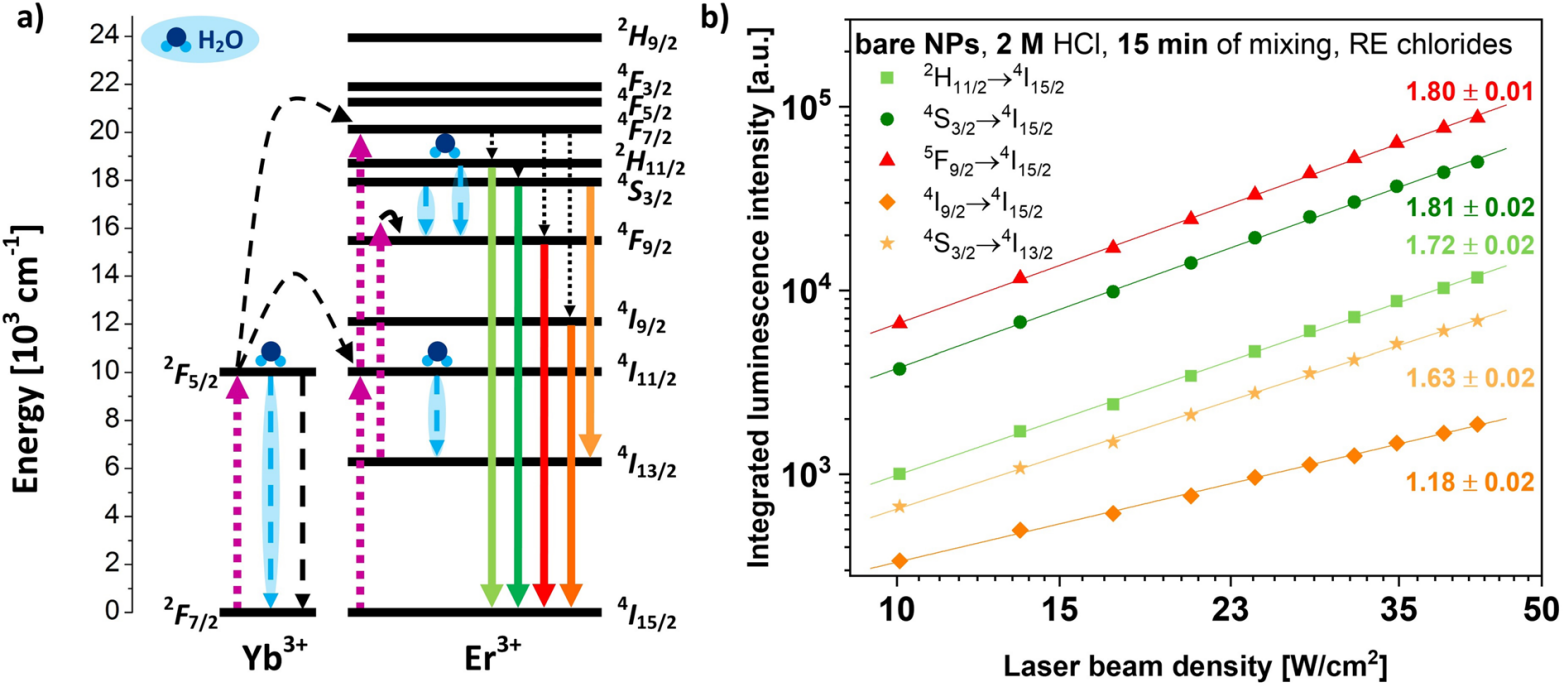
(**a**) Energy-level diagram for Yb^3+^ and Er^3+^ ions as well as proposed up-conversion mechanism (including de-excitation by water molecules) and (**b**) power-dependent slope values for Er^3+^ transitions in ligand-free NaYF_4_:Yb^3+^,Er^3+^/NaYF_4_ obtained from RE chlorides and modified for 15 min by using 2-M HCl solution.

The energy-level diagram of the used dopant ions Yb^3+^/Er^3+^, as well as their excitation and deactivation pathways, is presented in Fig. 5a. One possible route of excitation was based on the energy transfer UC mechanism, which took place between the dopant ions. The Yb^3+^ ions, which were sensitizers, absorbed the energy and were excited from the ground state to the ^2^F_5/2_ level. Then, the neighboring Er^3+^ ions called activators could be excited to the ^4^I_11/2_ level by the energy transfer from the Yb^3+^ ions. Another photon could be absorbed, resulting in the subsequent Er^3+^ excitation to the ^4^F_7/2_ level. Consequently, it was possible to observe non-radiative relaxation to the ^2^H_11/2_, ^4^S_3/2_, ^4^F_9/2_, and ^4^I_9/2_ levels (dotted arrows), as well as the UC emission (solid arrows) responsible for the different luminescence colors. Another possible excitation route was the excited-state absorption mechanism occurring from the ^4^I_13/2_ to the higher ^4^F_9/2_ Er^3+^ state. This route, as well as the energy transfer assisted by the Yb^3+^ ions, led to the enhancement of the red emission related to the feeding of the ^4^F_9/2_ level. Note that in the case of the NPs dispersed in an aqueous solution, the solvent played a critical role in the reduction of the UC luminescence intensity of the NPs^9^. The water quenching (wavy arrows) affected all the energy levels involved in the NIR to the visible UC mechanism. This could be attributed to the absorption of water near 980 nm, which could debilitate the excitation light^21^. Additionally, the water molecules could extract energy from the excited dopant ions. The energy gap between the ^4^I_11/2_ and the ^4^I_13/2_ levels of Er^3+^ well matched with the hydroxyl phonon energy (~ 3400 cm^–1^). Because of this fact, the ^4^I_11/2_ → ^4^I_13/2_ relaxation could be intensified through a cross-energy transfer to the water particles. The O–H vibrations also had an impact on the non-radiative decays of the ^4^S_3/2_ and the ^2^H_11/2_ levels of Er^3+^^35^.

In the UC process, the observed luminescence intensity (*I*_*UC*_) was proportional to the excitation laser power (*P*): *I*_*UC*_* ∝ P*^*n*^, where the *n* symbol represents the number of photons involved in the UC mechanism^36^. Therefore, the dependencies of the integral luminescence intensity on the laser beam density (Fig. 5b) were studied for the water-stable UCNPs characterized by the brightest UC emission. The UC emission intensities were measured using the 975-nm excitation wavelength and a laser beam density ranging from 10 to 43 W/cm^2^. Linear fitting with high coefficients of determination *R*^*2*^ (≤ 0.998) allowed us to calculate the slope values for five Er^3+^ electronic transitions: from ^2^H_11/2_, ^4^S_3/2_, ^5^F_9/2_ and ^4^I_9/2_ to ^4^I_15/2_, as well as from ^4^S_3/2_ to the ^4^I_13/2_ level. They were in the range of one to two (err ≤ 0.02), suggesting a two-photon excitation process, which was consistent with the proposed UC mechanism (Fig. 5a). Note that the observed slope values were not ideal integers. This phenomenon was related to the presence of various excitation possibilities, as well as the non-radiative decays of the Er^3+^ intermediate excited states^37^.

**Figure 6:**
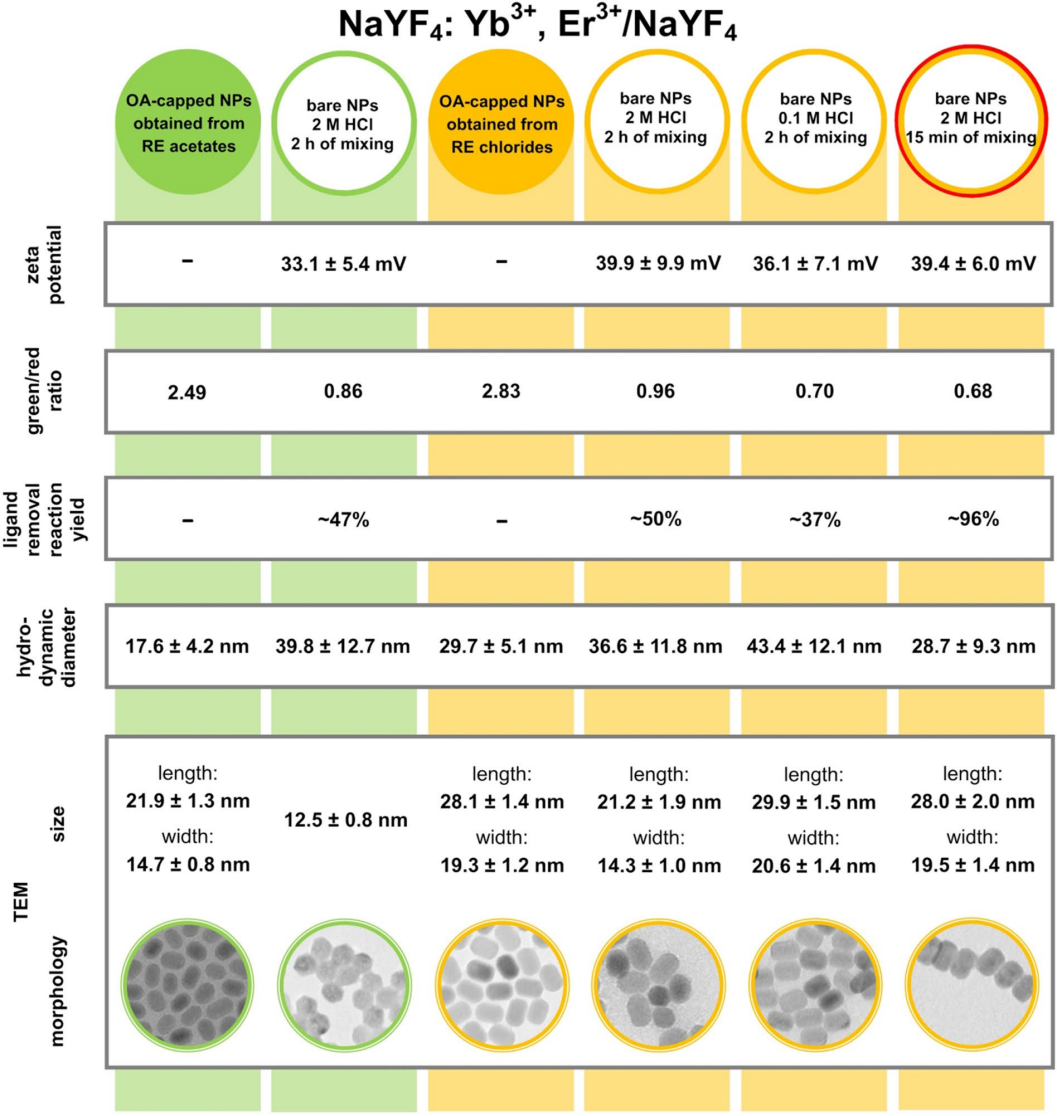
Overview of the zeta potential measurements, green-to-red emission ratios, surface modification yields, DLS measurements, and TEM analyses for both the OA-capped and the bare NaYF_4_:Yb^3+^,Er^3+^/NaYF_4_ particles. Color coding is used to indicate the UCNPs obtained from the RE acetates (green) and the RE chlorides (orange). The ligand-free product with the best properties was marked with the additional red circle.

By comparing three different combinations of ligand-free modification conditions, we could clearly distinguish the best approach for removing the organic ligands from the NPs’ surface. The above-described results of the zeta potential measurements, the green-to-red emission ratios, surface modification yields, DLS measurements, and TEM analyses for both the OA-capped and the bare NaYF_4_:Yb^3+^,Er^3+^/NaYF_4_ particles are shown in Fig. 6 to help the reader distinguish the differences between the modified crystals. Additionally, the ligand-free NPs characterized by the best physicochemical properties and the highest ligand removal reaction yield were marked with the extra red circle.

## Conclusions

To conclude, an improved procedure for obtaining the ligand-free NaYF_4_:Yb^3+^,Er^3+^/NaYF_4_ UCNPs was developed. Three different combinations of modification conditions based on the changes in the acid molarity and the mixing time were tested to confirm their impact on the physicochemical properties of the NPs. The hexagonal OA-capped crystals were synthesized using precipitation in high-boiling-point solvents. Additionally, the organic ligand removal was performed successfully by using the HCl solution. The procedure based on the use of the 2-M HCl solution and 15 min of mixing was the most effective of all of the tested approaches, as it enabled us to achieve the highest reaction yield of up to 96%. Importantly, in comparison to those of the hydrophobic NPs, the size and the shape of the bare crystals modified according to this approach remained unchanged. The small UCNPs were stable in the water solution even after six months, which made it possible for us to apply them in biology and medicine. The materials exhibited visible UC emission under 975-nm excitation, which confirmed the efficient energy transfer between the used dopant ions.

## Supplementary Information

Supplementary Information.
