# Supplementary material for: Improvement of ligand-free modification strategy to obtain water-stable up-converting nanoparticles with bright emission and high reaction yield
Source: Sci Rep. 2021 Sep 22;11:18846. doi: 10.1038/s41598-021-98240-0 (PMC8458358; doi:10.1038/s41598-021-98240-0)
Supplement: Supplementary file 1 — Supplementary Information. [file 41598_2021_98240_MOESM1_ESM.pdf]

Supporting Information

for

Improvement of ligand-free modification strategy to obtain water-stable up-converting nanoparticles with bright emission and high reaction yield

Natalia Jurga, Dominika Przybylska, Piotr Kamiński, and Tomasz Grzyb*

Department of Rare Earths, Faculty of Chemistry, Adam Mickiewicz University in Poznań, Uniwersytetu Poznańskiego 8, Poznań 61-614, Poland

*Correspondence: tgrzyb@amu.edu.pl

Contents:

1. Synthesis description
2. Figure S1. (DLS measurements)
3. Figure S2. (FT-IR spectra)

1. Synthesis description

1.1. Synthesis of oleate-capped $NaYF_4$:Yb^{3+},Er^{3+}/$NaYF_4$ core/shell UCNPs using RE acetates

The UCNPs were obtained according to the procedure described by Rinkel *et al.* [1] with some modifications.

1.1.1. Synthesis of anhydrous rare earth acetates

RE oxides Y_2O_3, Er_2O_3, and Yb_2O_3 were used to obtain the appropriate RE anhydrous acetates. The appropriate RE oxide, acetic acid, and distilled water (1 mmol; 4 ml; 4 ml, respectively) were placed in a three-necked round-bottom flask and heated at 95°C in a reflux condenser. After the dissolution of the RE oxide, the mixture was cooled to room temperature. Subsequently, the solvent was evaporated with a rotavapor to almost a dry powder, to which the appropriate amount of acetic acid (1.5 ml of acid on 1 mmol of RE) was added and stirred at 60°C. The dissolved RE hydrous acetate was transferred to a three-necked round-bottom flask with a dropping funnel and heated to 80°C in a reflux condenser. Then, the appropriate amount of anhydrous acetic acid (1.5 ml of acid on 1 mmol of RE) was dropped via the dropping funnel to the solution, and the temperature was maintained above the boiling point of glacial acetic acid (around 120°C) for 2 h. Next, the mixture was cooled to room temperature, and the solvent was removed with the rotavapor to obtain a dry powder. The residual water content in the final product was determined by a thermogravimetric analysis (TGA).

1.1.2. Synthesis of α-$NaYF_4$:Yb^{3+},Er^{3+} cores

The mixture of high-boiling-point solvents (oleic acid and 1-octadecene) was placed into a three-necked round-bottom flask with a thermosensor, connected to the Schlenk line, and outgassed for 60 min at 100°C (the heating rate was increased by 1°C every minute, starting from room temperature) under vacuum ($<10^{-1}$ mbar). The solution was cooled down to room temperature, and anhydrous RE acetates were added in the right ratio (Y^{3+}:Yb^{3+}:Er^{3+}, 0.8:0.18:0.02, respectively). Thereafter, the solution was outgassed under vacuum ($<10^{-1}$ mbar) at room temperature and heated up to 100°C for 60 min again. Next, sodium oleate (in the ratio of 3:1 to

RE ions) was placed into the hot solution under the flow of nitrogen and degassed to complete dissolution. Later, ammonium fluoride (6 mmol per mmol of RE ions) was added, and the apparatus was cycled three times between vacuum and nitrogen for 5 s on each cycle. The mixture was heated to 200°C (the heating rate was increased by 1°C every minute, starting from room temperature) under the flow of nitrogen and kept at this temperature for 60 min. Thereafter, the solution was cooled to room temperature, and the obtained suspension was centrifuged to remove the sodium fluoride by-product. Finally, the particles were precipitated from the supernatant by adding ethanol (1 ml of ethanol per 1 ml of supernatant) and collected by centrifugation as well as washed with n-hexane. The precipitate was dried at room temperature. The purity of the product was checked using TGA.

1.1.3. *Synthesis of β-$NaYF_4$:Yb^{3+},Er^{3+} cores*

The α-$NaYF_4$:Yb^{3+},Er^{3+} core precursor was mixed with high-boiling-point solvents (oleic acid and 1-octadecene, in a 1 mmol α-core:4 ml OA:4 ml ODE ratio), placed in a three-necked round-bottom flask with a thermosensor, and connected to the Schlenk line. The mixture was degassed under vacuum ($<10^{-1}$ mbar) at room temperature and kept at 100°C (the heating rate was increased by 1°C every minute, starting from room temperature) for 90 min. Next, the solution was heated to 300°C (the heating rate was increased by 1°C every minute, starting from room temperature) in a nitrogen flow for 55 min and cooled down. The obtained solution was mixed with n-hexane and ethanol in an equal volume ratio and centrifuged. The product was purified with n-hexane and precipitated after the addition of ethanol and centrifuged. Finally, the NPs were dried at room temperature.

1.1.4. *Synthesis of α-$NaYF_4$ shell precursor*

The synthesis of the α-shell precursor was analogous to the preparation of the α-core described earlier. Each change that was made is marked in this paragraph. In the synthesis of the α-$NaYF_4$ shell, only anhydrous yttrium acetates were used out of the mixture of the RE ions. Additionally, the ratio of sodium oleate to RE ions changed to 1.5:1.

1.1.5. Synthesis of β-$NaYF_4$:Yb^{3+},Er^{3+}/β-$NaYF_4$ core/shell NPs

The core particles and the shell precursor (core-to-shell ratio of 1:7) were added to the mixture of high-boiling-point solvents (oleic acid and 1-octadecene, 1 mmol β-core + α-shell:2 ml OA:2 ml ODE) and outgassed by stirring under low pressure (<10^{-1} mbar) at 100°C (the heating rate was increased by 1°C every minute, starting from room temperature) in a three-necked round-bottom flask for around 90 min. Next, the solution was heated at 300°C (the heating rate was increased by 1°C every minute, starting from room temperature) for 135 min and cooled down. The obtained solution was mixed with n-hexane and ethanol and centrifuged (5 min, 9000 rpm). Subsequently, the precipitate was dissolved in n-hexane, the same amount of ethanol was added, and the precipitate was centrifuged. Finally, the NPs were dispersed in n-hexane and stored at 4°C.

1.2. Synthesis of oleate-capped $NaYF_4$:Yb^{3+},Er^{3+}/$NaYF_4$ core/shell UCNPs using RE chlorides

The core/shell UCNPs based on RE chlorides were obtained using the modified procedure described by Gerelkhuu *et al.* [2]. Each synthesis step was analogous to the procedure described in Sections 1.1.2–1.1.5 with a minor change in the increase in the outgassing time during the preparation of both the β-core and the core/shell NPs to 120 min.

The synthesis of the chloride RE precursor as another source of RE ions is described below.

1.2.1. Synthesis of rare earth chlorides

The RE oxide (Y_2O_3, Er_2O_3, and Yb_2O_3) was dissolved in the mixture of demineralized water and hydrochloric acid (25 ml H_2O and 5 ml of HCl was used per 1 mmol of RE_2O_3) to synthesize the appropriate RE chloride. The solution was kept at 70°C overnight and stirred in a reflux condenser. Thereafter, a clear solution was evaporated under vacuum by using a rotary evaporator. Finally, the RE chlorides were dried at 110°C overnight under atmospheric pressure.

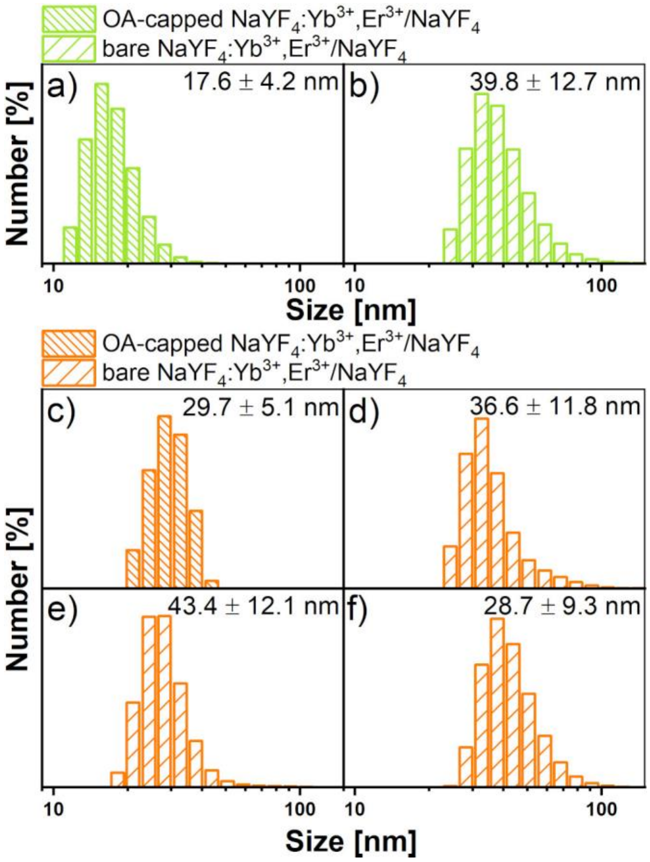

Figure S1. Hydrodynamic diameter and size distribution profile in DLS study of (a, c) OA-capped and (b, d, e, f) ligand-free $NaYF_4$:Yb^{3+},Er^{3+}/$NaYF_4$ UCNPs. Different modification conditions were used (b, d) 2 M/2 h, (e) 0.1 M/2 h, and (f) 2 M/15 min, which denote HCl molarity and time of mixing, respectively. Color coding is used to indicate the UCNPs obtained from the RE acetates (green) and the RE chlorides (orange).

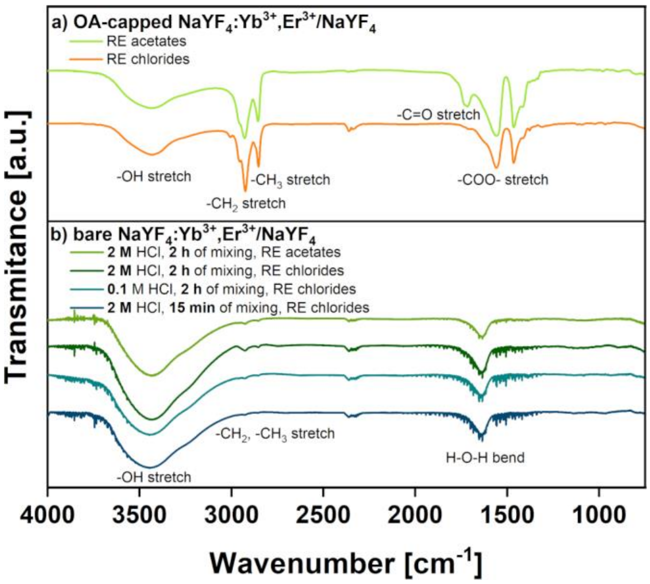

Figure S2. FT-IR spectra of (a) OA-capped and (b) bare $NaYF_4$:Yb^{3+}, Er^{3+}/$NaYF_4$ UCNPs.

References:

[1] Rinkel, T., Raj, A. N., Dühnen, S. & Haase, M. Synthesis of 10 nm β-$NaYF_4$:Yb,Er/$NaYF_4$ Core/Shell Upconversion Nanocrystals with 5 nm Particle Cores. *Angew. Chemie* **128**, 1177–1181 (2016)

[2] Gerelkhuu, Z. *et al.* One-step synthesis of $NaLu_{80-x}Gd_xF_4$:Yb_{18}^{3+}/Er_2^{3+}(Tm^{3+}) upconversion nanoparticles for in vitro cell imaging. *Mater. Sci. Eng. C* **86**, 56–61 (2018)
